# Microstructure Evolution and Mechanical Properties of PM-Ti43Al9V0.3Y Alloy

**DOI:** 10.3390/ma13010198

**Published:** 2020-01-02

**Authors:** Dongdong Zhang, Na Liu, Yuyong Chen, Guoqing Zhang, Jing Tian, Fantao Kong, Shulong Xiao, Jianfei Sun

**Affiliations:** 1 School of Materials Science and Engineering, Harbin Institute of Technology, Harbin 150001, China; 2 Beijing Institute of Aeronautical Materials, Beijing 100095, China; 3 College Vanadium and Titanium, Panzhihua University, Panzhihua 617000, China

**Keywords:** TiAl powder, HIP, fine grain, submicron precipitate

## Abstract

A novel strategy of microstructure design is introduced to improve the mechanical properties of TiAl alloys, fabricated by powder metallurgy. The gas atomization powder and as-HIPed (Hot isostatic pressing) TiAl are investigated by scanning electron microscopy, energy dispersive spectrometry, transmission electron microscopy, and electron backscattered diffraction. The dispersed submicron precipitate in the microstructure is determined to be Y_2_O_3_. A microstructure with uniform fine grain is obtained. The room temperature strength and strain reach 793 MPa and 1.5%, respectively. The strength and strain at 700 °C are still as high as 664 MPa and 9.2%, respectively. The fine grain and precipitate lead to a high room-temperature plasticity.

## 1. Introduction

TiAl alloys are a new type of promising lightweight material [1,2,3]. The TiAl alloys could be a good substitute for superalloys due to their low density, high specific strength, high Young’s modulus, and oxidation resistance at 700–900 °C [4,5]. The Ti-48Al-2Cr-2Nb alloy developed by GE has now been successfully applied to the last two-stage low-pressure turbine blades of the GEnx-1b engine [6]. However, aero-engine turbine blades face high-temperature, complex-stress working environments, which place high requirements on the high-temperature strength and high-temperature durability of materials [7]. The TiAl produced by the traditional casting method and ingot metallurgy method have coarse grains and a non-uniform microstructure, which is disadvantageous for the high-temperature performance of TiAl alloys. TiAl alloys via powder metallurgy (PM) result in outstanding mechanical properties, owing to their uniform and fine microstructure [8]. Gerling used HIP (Hot isostatic pressing) to sinter and densify TiAl powder, and obtained a dense and non-porous microstructure [9]. Liu prepared TiAl billet by HIP, at 1250 °C/150 MPa/5 h, and the TiAl billet reached approximately 600 MPa and 0.55%, respectively [10]. However, the O content of PM TiAl is generally higher than that of casting and forging TiAl. The high O content would degenerate plasticity [11]. Many methods, including micro-alloying and high-vacuum experimenting, have been used to reduce the O content of TiAl alloys [12,13]. TiAl powder needs to be degassed in a vacuum before HIP. Gerling degassed TiAl powder at 500 °C/0.003 Pa [9]. Liu degassed TiAl powder at 500 °C in a vacuum for 12 h [10]. Existing studies have shown that rare earth addition could not only reduce the O [14], but also refine grain. Y [15,16] could purify the matrix and increase the plasticity of TiAl alloys. The dispersed Y-rich phase could effectively refine the grain, because it can act as a heterogeneous nucleus. Trivedi investigated the Ti-48Al-2Cr-2Nb alloy with Y addition by mechanical alloying and found that grain growth was inhibited, and ductility or fracture toughness was maintained [17]. Adding Y into the TiAl alloy could reduce the O content of the matrix, thereby forming the particulate oxide. The PM could prepare fine grain TiAl alloys. Thus, combining the Y addition and PM method might result in high-plasticity precipitate-reinforced TiAl alloys.

In the present study, the microstructural development and mechanical properties of the submicron precipitate-reinforced β-TiAl alloys produced by PM are investigated.

## 2. Experimental Procedures

### 2.1. Fabrication of Powder

The powder of the TiAl alloys was produced by electrode induction melting gas atomization (EIGA) under inert gas. The nominal chemical composition of the powder in this study was Ti-43Al-9V-0.3Y (at%). A pre-alloyed ingot was produced by vacuum arc melting. The ingot was melted threefold to ensure a homogenous distribution and then the ingot was used in EIGA. The entire atomization process was performed in a high-purity Ar atmosphere. The molten TiAl flowed in a special nozzle and was atomized into a nearly spherical micron powder by an inert gas flow with a sonic velocity. The hollow powder and gas content increased with the expansion of the particle size of the atomized powder. This condition would seriously affect the mechanical properties. The powder observation result manifested that the powder defect with a particle size of ≥100 μm or more was significantly increased. Thus, the powder with a particle diameter of ≤80 μm was chosen.

### 2.2. Hot Isostatic Pressing

The pre-alloyed TiAl powder was poured into a 304 stainless steel can with a size of Φ130 × 135 mm. The steel thickness was 2 mm. The can was sealed and degassed at 400 °C/10^−3^ Pa for 8 h. HIP was performed in an Ar atmosphere at a temperature of 1200 °C and a pressure of 140 MPa for 5 h with a QIH32 HIP furnace (Allmanna Svenska Electriska Aktiebolaget Corporation, Vasteras, Sweden ).

### 2.3. Microstructural Characterizations

The microstructure and chemical composition of the powder and billet were observed with a Quanta 200FEG field emission scanning electron microscope (SEM, Field Electron and Ion Company, Hillsboro, OR, USA) equipped with an energy dispersive spectrum (EDS, Field Electron and Ion Company, Hillsboro, OR, USA) detector. Crystallographic analysis was conducted by electron backscatter diffraction (EBSD, TSL OIM Analysis 6, Hillsboro, OR, USA). Precipitates and dislocations were observed by Talos F200X transmission electron microscopy (TEM, Field Electron and Ion Company, Hillsboro, OR, USA). The specimens for SEM and EBSD were prepared by successive mechanical polishing with SiC papers up to 2000 mesh (Mingniu Abrasive Materials Co., Ltd., Foshan, China). Thereafter, the specimens were electro-polished in a solution of 10% perchloric acid, 30% butanol, and 60% methanol at −25 °C and 30 V. The TEM samples were obtained by ion thinning. O and N content was measured using a LECO GDS850A glow discharge spectrometer (LECO Corporation, San Jose, CA, USA).

The SEM-EDS is an important and effective method to determine the phase composition by the elemental content. The X-ray intensity of a specific element excited by an electron beam is positively correlated with its elemental content. Comparison results of the intensity of each X-ray indicate that the composition ratio of various elements could be obtained. However, the accuracy of the EDS results is affected by many factors, such as the atomic number effect (Z), the X-ray absorption effect in sample (A), and the fluorescence effect (F). The phase size would also affect the accuracy of the EDS results. The precipitates in this work were fine, and their sizes were submicron and micron. The precipitates required a ZAF calibration method to calibrate the EDS results. According to Criss and Birks [18], the EDS results require a threefold iteration to be calibrated to the appropriate accuracy. The iteration equation is as follows:

(1) $$C_{m}=\frac{k_{m}C\left(1-k\right)}{k_{m}\left(C-k\right)+k\left(1-C\right)}$$

K = C × (Z × A × F)

where 

*C_m_* is the elemental content iteration value;

*C* is the initial elemental content;

Z, A, F, and k_m_ are the values given by the EDS instrument.

### 2.4. Mechanical Property Tests

The tensile tests were performed on Instron5569 (Instron Corporation, Canton, NY, USA) and AG-X Plus material testing machines (Shimadzu, Kyoto, Japan) at room temperature and 700–900 °C. The tensile speeds were 1 × 10^−4^/s and 5 × 10^−4^/s for room and high temperatures, respectively. The specimens were prepared by successive mechanical polishing with SiC papers up to 2000 mesh. Before the high-temperature tensile tests, the specimens were heated at a specific high temperature for 5 min. 

## 3. Results and Discussion

### 3.1. Characterizations of Powder

**Figure 1:**
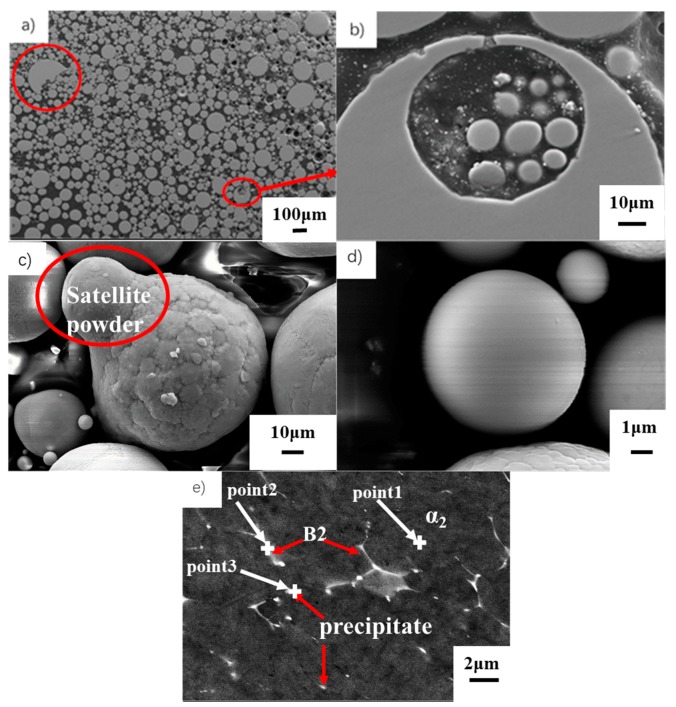
Morphology and microstructures of TiAl powder: (**a**) overview of TiAl powder; (**b**) a powder particle containing inner pore and small particles; (**c**) dendritic powder particle connected to a smooth satellite particle; (**d**) smooth powder; (**e**) powder microstructure at BSE (back scatter) mode.

Figure 1 shows the powder morphology and microstructure. The powder is almost perfectly spherical. A satellite or hollow powder is difficult to find. In the high-speed inert gas flow, a small amount of molten TiAl droplets would collide and become semi-spherical (red circle in Figure 1a). In the EIGA of TiAl alloys, the Ar gas would still react with a small amount of molten TiAl, and a small amount of gas would be trapped inside the droplet. After cooling and solidification, the droplet becomes a hollow powder (Figure 1a). Figure 1b shows an enlarged image of the red circle in Figure 1a. The small powder is wrapped in a hole. The large powder surface shows a dendrite morphology (Figure 1c). The small powder owns a smooth surface (red circle in Figure 1c). No apparent feature can be observed on the surface (Figure 1d). The particle size of the TiAl pre-alloyed powder in this work ranges from 2.2 μm to 160 μm. The powder particle size varies greatly. The precipitate size in Figure 1d is approximately 8 μm, whereas that in Figure 1e is 10–100 μm and is the internal microstructure. Thus, Figure 1d,e show extremely different results.

Many researchers have found that the V is one of the β phase stabilizers [19,20]. Su et al. found a type of white network of the B2 phase in the forged TiAl containing a high V [21]. The gray phase is the α_2_ phase, and the white linear phase is B2 (Figure 1e). The powder mainly consists of α_2_, and only contains trace B2 and white precipitate (Figure 1e). This manifestation indicates that the cooling speed might be the main factor in changing the microstructure.

Ke et al. found that Y would combine with Al to form a linear or network Al_2_Y in casting TiAl alloys [22]. In general, PM TiAl alloys contain high O. Y easily reacts with O [23,24,25]. According to Table 1, the precipitate contains a high content of Y and O elements, and Y/O is close to 2/3. Thus, the white precipitate might be Y_2_O_3_. The small diameter closed pore is still inside the powder. The powder particle size statistics indicate that powder with a diameter below 80 μm accounts for 90% and the average diameter is 38 μm. 

### 3.2. As-HIPed Microstructures

**Figure 2:**
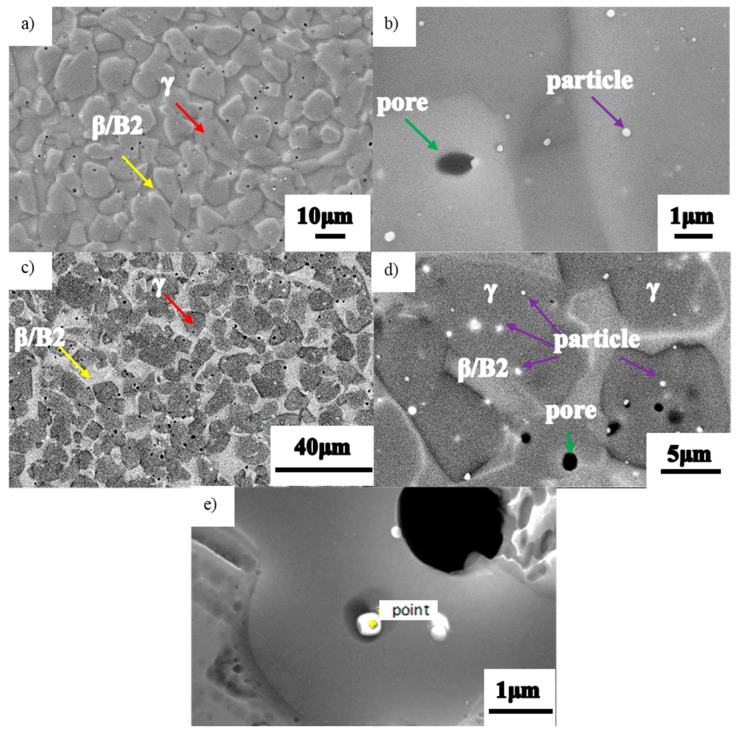
Microstructures of as-HIPed TiAl alloy billet: (**a**) low- and (**b**) high-magnification images in secondary electronic mode; (**c**) low- and (**d**) high-magnification images in backscattered electron mode; (**e**) submicron precipitate.

Figure 2 shows the as-HIPed microstructure. The TiAl billet has a dual-phase microstructure, and primary particle boundaries are obscured. The dark phase is the γ phase and the gray one is B2 (Figure 2c,d). The γ phase is rather substantial. After electrolytic polishing, B2 substantially dissolves and the γ exhibits a surface convex feature (Figure 2a). The SEM images show that the γ grains are uniformly dispersed and the B2 one surrounds these island-like γ grains (Figure 2a,c). A small black pore (Figure 2b,d,e) and distributed submicron white precipitate also exist. This appearance might be due to the following: Micron- and nano-sized Y_2_O_3_ precipitates are dispersed in the microstructure of as-HIPed TiAl alloy. During electropolishing, the electrode potential of these precipitates differ from the surrounding matrix. The joint between the precipitate and the matrix is rapidly corroded. Consequently, part of the precipitates fall off the matrix and form small holes in the microstructure.

The volume fraction of precipitates in the powder is about 0.7%. The volume fraction of precipitates in as-HIPed TiAl is about 2.2%. Therefore, The quantity of the precipitates increases compared with that of the powder. Most pores and precipitates are distributed in the γ phase or γ phase edges (as indicated by the green and purple arrows in Figure 2b,d). The possible trace elements, such as C and N, are excluded for the following reasons: (1) The N content is low. In this work, the N and O contents are 140 ppm and 760 ppm, respectively. Such a low N content has difficulty forming a precipitate in TiAl. (2) N exhibits difficulty in reacting with TiAl and is completely dissolved in the matrix at a low content compared with O. The solid solution and precipitation effects are negligible. Although C reacts with molten TiAl alloy to form trace amounts of Ti_2_AlC and Ti_3_AlC, it is a highly stable element for TiAl alloys at high temperature. Thus, graphite could be used as a crucible material for the smelting and casting of TiAl alloys. During the atomization and HIP process, the entire material preparation process is conducted under a high vacuum/Ar atmosphere. Thus, the C element, or other contaminant ones in the environment, has almost no effect on the material. Therefore, these trace elements are unanalyzed. The literature reported that a TiAl alloy containing 0.3% (at%) of Y element evidently contains a large amount of Y-containing precipitates in the microstructure [26,27,28]. In Figure 1e and Figure 2d, fewer precipitates in the powder microstructure and considerable precipitates in the as-HIPed microstructure can be observed. In the atomization process, the metal stream is broken into a micron-sized spherical metal droplet under the impact of a high-pressure inert gas stream. Subsequently, micron-sized metal droplets are rapidly cooled. Most Y elements exhibit delayed precipitation out of the matrix, because of the fast cooling. Only a small amount of precipitate is left in the powder. During HIP, the Y element slowly precipitates from the matrix at a high temperature, eventually leaving a large amount of precipitates in the microstructure. 

The point EDS results for the submicron precipitate show that it contains approximately 37.54% Y and 59.58% O (at%). Trivedi et al. found that the Y would form a type of oxide containing Y, Al, and O in Ti-48Al-2Cr-2Nb [29]. These fine precipitates could significantly refine the grain and improve the creep resistance of the material [30]. The EDS manifests that the atomic ratio of Y/O is about 2/3 and the white submicron precipitate might be identified as Y_2_O_3_.

### 3.3. EBSD Investigation

**Figure 3:**
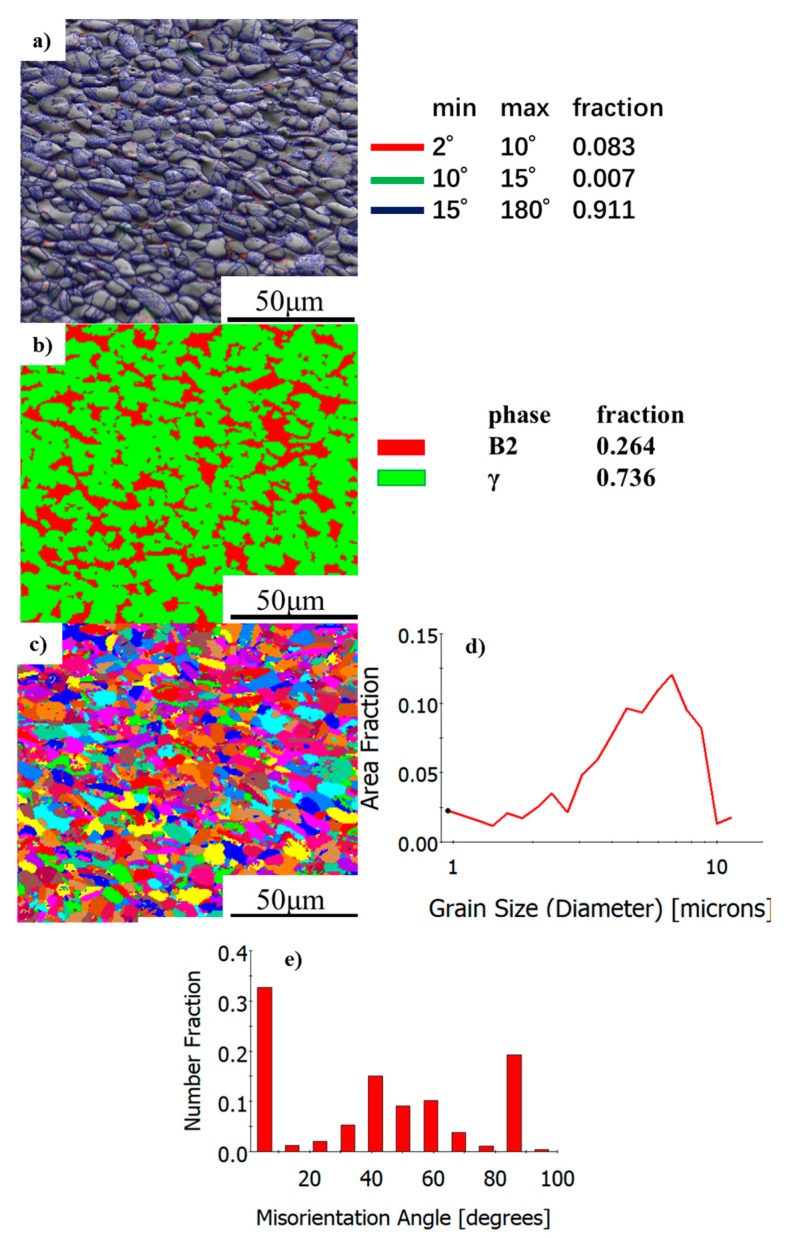
EBSD map of as-HIPed TiAl alloy billet: (**a**) LAGBs, MAGBs, and HAGBs; (**b**) phase distribution; (**c**) grain graphics; (**d**) grain size distribution; (**e**) misorientation angle distribution.

Figure 3 shows the EBSD maps of the as-HIPed TiAl billet. The grain boundary could be divided into three types on the basis of its orientation angle. The types are low- (LAGB, 2°–10°), medium- (MAGB, 10°–15°), and high- (HAGB, 15°–180°) angle grain boundaries. The low-angle grain boundary is usually caused by dislocation. MAGB is considered as a prerequisite for the nucleation of continuous dynamic recrystallization, which is associated with the accumulation and tangle of dislocation. The HAGB is formed due to the nucleation-growth mechanism. In Figure 3a, the LAGB, MAGB, and HAGB account for 8.3%, 0.7%, and 91%, respectively. During the HIP, the powder sintering and densification are accompanied by plastic deformation. Hereafter, the gap between the powders is filled. Grain re-growth is accompanied by a solid phase transformation. The α in the powder is transformed to γ and β phases. After cooling to room temperature, fine equiaxed grain (γ + B2) is formed. This condition leads to the high misorientation angle in Figure 3c. γ and B2 account for 73.6% and 26.4%, respectively, and are uniformly dispersed. B2 has a bcc (Body-centered cubic) crystal structure with 12 slip systems, and is easily deformed at high temperatures. Thus, B2 could act as a softening phase to promote deformation.

Grain boundary sliding plays an important role in superplastic deformation. The fine grain could decrease the stress concentration, which is beneficial to the subsequent hot working deformation, such as forging and rolling. The orientation angular difference distribution shows a W-shape, with peaks on both sides and the middle, and two troughs at 20° and 80°. The grain orientation difference is only a small part within 10° in Figure 3e. This notion indicates that the substructural part in the microstructure is small [31,32,33]. Huizhong Li obtained TiAl alloy billets by using TiAl powder and HIP. The microstructure of the as-HIPed Ti-45Al-7Nb-0.3W alloy showed a near-γ microstructure with a mean grain size of about 12 μm [34] It is reported that the α phase and β phase followed the Blackburn orientation relationship (BOR) by EBSD analysis. In Figure 3b, the as-HIPed TiAl shows a dual-phase microstructure, consisting of α phase and β phase. In Figure 1e, the microstructure is composed of α phase. The microstructure is very fine and uniform, the grain diameter is about 7 μm. A large number of γ phases formed by HIP have good plasticity, which is beneficial to the plasticity of the TiAl.

### 3.4. Mechanical Behaviors

**Figure 4:**
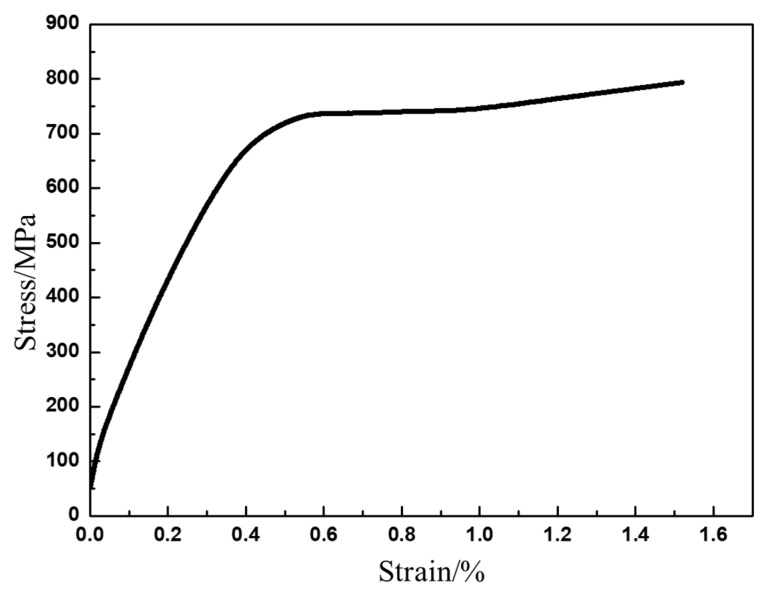
Tensile stress–strain curve of as-HIPed Ti43Al9V0.3Y at room temperature.

**Figure 5:**
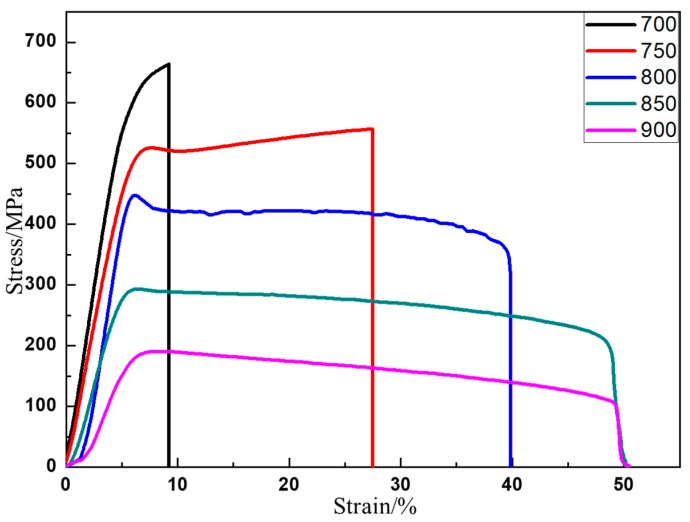
Tensile stress–strain curve of as-HIPed Ti43Al9V0.3Y at high temperatures (°C).

The room temperature stress–strain curve of as-HIPed TiAl is shown in Figure 4. It shows that TiAl still undergoes a large amount of plastic deformation after the elastic deformation stage. The room temperature plastic elongation to fracture is 1%. The YS is approximately 669 MPa and the UTS is roughly 793 MPa (Table 2). After yielding, the stress still increases by more than 100 MPa, which might be due to the Y_2_O_3_ (Figure 2e) hindering the dislocation slip [35]. At 700 °C, the UTS is 664 MPa and the strength remains at a high level and shows no evident decrease, however, the strain significantly increases by more than 9.2% (Table 2). The dislocation is hindered by a submicron Y_2_O_3_ precipitate (Figure 7). The dislocation would accumulate to form a high-density dislocation plugging network, thereby preventing plastic deformation and ultimately improving the strength [35,36,37,38,39,40,41,42]. From 700 °C to 750 °C, the strain rapidly increases from 9.2% to 27.4% (Table 2 and Figure 5), indicating that the microstructure significantly changes. The strength decreases by approximately 100 MPa to 150 MPa, with the temperature increasing by 50 °C. Above 850 °C, the plasticity almost shows no increase, but the strength drastically decreases (Table 2 and Figure 5). From room temperature to 750 °C, the stress slowly increases with the strain increase after yielding. However, between 800 °C and 900 °C, the stress slowly decreases with the increase of strain after yielding (Table 2 and Figure 5).

Table 3 presents a comparison of the TiAl alloy properties and shows that the TiAl alloy in this work has good plasticity at room temperature.

### 3.5. Study of the Fracture Surfaces

**Figure 6:**
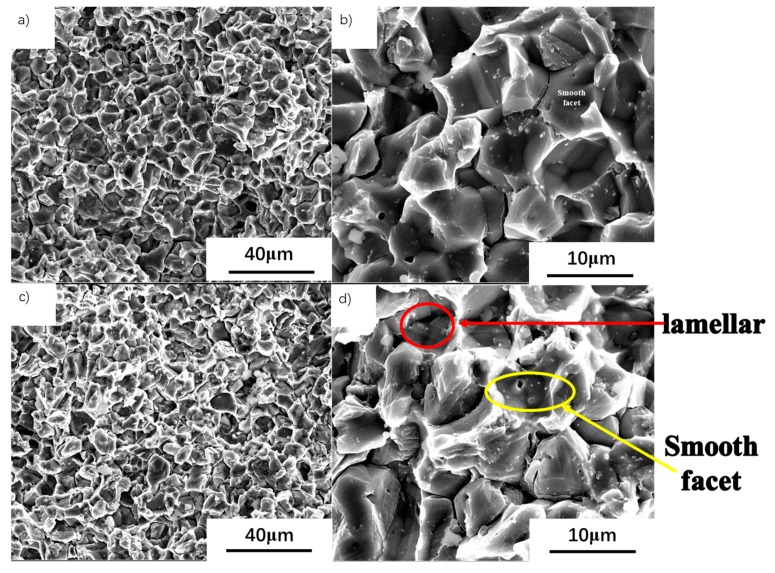
Fractural morphology of tensile specimens at (**a**,**b**) 700 °C and (**c**,**d**) 750 °C.

**Figure 7:**
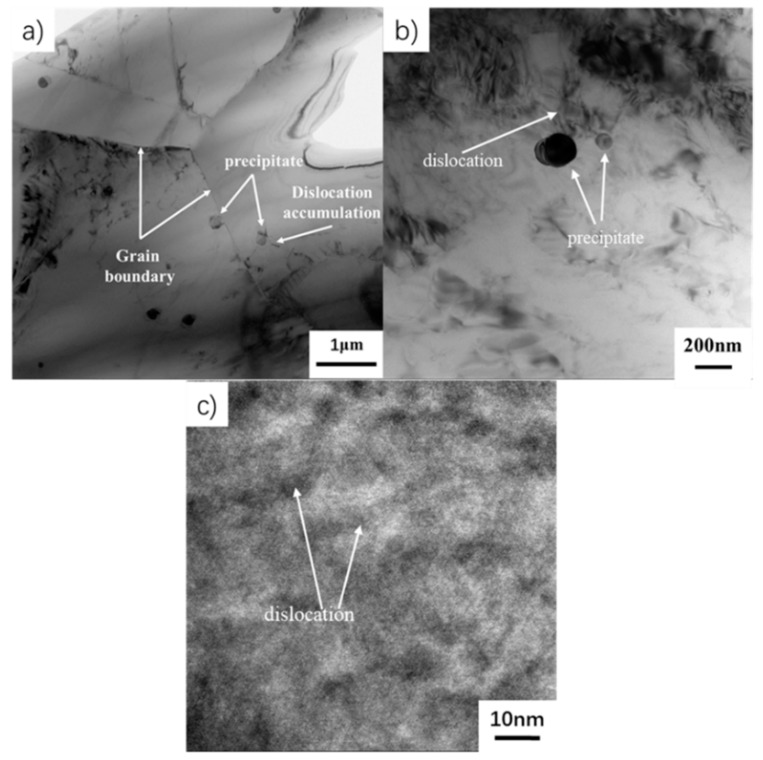
TEM of tensile specimen tested at 750 °C (**a**) grain and particle distribution (**b**) particles and dislocations (**c**) high resolution of dislocations in grains.

The fracture at 700 °C and 750 °C still exhibits brittle fracture characteristics (Figure 6). A little of the point-like precipitates is still on the surface. At 700 °C, the fracture is a sugar-like granular morphology, showing a distinct intergranular fracture. The fractured surface is very smooth and neat. And a small number of point-like white precipitates are on the fractured surface (Figure 6a,b). The precipitates are mostly distributed in the γ phase grain. Only a few precipitates are in the B2 and on the grain boundary (Figure 2b,d). The fractured surface consists of a regular geometric facet at 700 °C and a smooth facet is left after the grain cracks at the grain boundary (Figure 6b). By contrast, the fractured surface at 750 °C is rough (Figure 6d), and the facet has intergranular and transgranular features. In addition, a morphology similar to the lamellae is observed. At 700 °C, deformation is difficult inside the grain. Because the grain boundaries are soft relative to the inside of the grains, the deformation is mainly driven by the slip and deformation of the grain boundaries. The dislocation proliferates, slips, and accumulates in the vicinity of the grain boundary and surrounds the submicron precipitates. Dislocations contribute less to deformation. High stress forms around the grain boundary and precipitates. The crack occurs at the triangular grain boundary and expands along the grain boundary when it reaches critical stress (Figure 6). At 750 °C, the inside of the grain becomes soft relative to 700 °C, and large deformation can occur inside the grain. Together with the grain boundaries, it could also contribute to the deformation. The grains show more plastic behavior. Therefore, the grains in the fractured surface are elongated (Figure 6d). And at 700 °C, the grains are hard inside and the deformation is limited. The grains in the fractured surface still remain equiaxed without obvious elongation (Figure 6b). This shows that the hard-to-soft transition temperature inside the grain is between 700 °C and 750 °C. The dislocation slipping distance from the start to the precipitate is short, because the precipitates are small and uniformly distributed. The corresponding stress concentration is also small. Consequently, the crack expands along the grain boundary where the local stress is high. The precipitates distributed in the TiAl alloy microstructure are Y_2_O_3_. This result is obtained by indexing the diffraction patterns of the precipitates (Figure 7). And when a dislocation slip network is generated in the microstructure, the dislocation plugging is formed around the fine Y_2_O_3_ precipitate. In Figure 7b, nano-precipitates hinder the dislocation network. This situation would lead to the second-phase enhancement. 

## 4. Conclusions

The main conclusions are summarized as follows:

1. The TiAl powder prepared by gas atomization has high sphericity. The small powder is smooth and the large one exhibits a dendritic appearance. Although the alloy contains high β stabilizer V, the powder mainly consists of α_2_ due to the fast cooling rate.
2. The phase composition of as-HIPed TiAl and TiAl powder varies greatly. The microstructural evolution is as follows: α_2_ + trace B2 → γ + B2 from powder to billet. The microstructure is fine and uniform and the grain diameter is about 7 μm. Many γ phases and fine grain are beneficial to the plasticity of the TiAl.
3. TiAl is mainly deformed when the grain boundary slips below 700 °C. When the temperature reaches 750 °C, both TiAl grains and grain boundaries start to contribute to deformation. The hard-to-soft transition temperature inside the grain is between 700 °C and 750 °C. It is the reason that the plasticity of TiAl alloy is greatly improved between 700 °C and 750 °C.
4. The YS and UTS of the as-HIPed TiAl at room temperature are 669 MPa and 793 MPa, respectively. The plastic elongation to fracture is 1%. At 700 °C, the YS and UTS still reach 589 MPa and 664 MPa, respectively. Fine grain and submicron Y_2_O_3_ precipitates enhance the room-temperature and high-temperature strength and plasticity.

## Figures and Tables

**Table 1:** Point EDS results of powder in Figure 1e.

Select Points	Point 1 (at%)	Point 2 (at%)	Point 3 (at%)
Al	42.81	42.34	26.58
Ti	48.74	48.33	32.37
V	8.32	9.18	5.49
Y	0.05	0.08	13.51
O	0.08	0.07	22.05
Total	100.00	100.00	100.00

**Table 2:** Tensile properties of as-HIPed Ti-43Al-9V-0.3Y at room and high temperatures.

	RT	700 °C	750 °C	800 °C	850 °C	900 °C
σ_s_ (MPa)	669 ± 23	589 ± 12	505 ± 21	440 ± 15	279 ± 8	172 ± 6
σ_b_ (MPa)	793 ± 35	664 ± 20	556 ± 29	448 ± 10	292 ± 7	190 ± 5
strain (%)	1.52 ± 0.17	9.2 ± 0.6	27.4 ± 1.1	39.5 ± 1.3	48.4 ± 1.6	49.2 ± 1.1

**Table 3:** Tensile properties of TiAl alloys in the literature and in this work [24,25,42].

Alloys		Room Temperature		700 °C		
		UTS (MPa)	Strain (%)	UTS (MPa)	Strain (%)	
Ti-43Al-9V-0.2Y	rolling	826	1.4	674	27.1	
Ti-43Al-9V-0.2Y	casting	561–634	0.45–0.76	/	/	
Ti-43Al-2Cr-2Mn-0.2Y	forging	657	0.86	496	10	
Ti-43Al-9V-0.3Y	as-HIPed	793	1.5	664	9.2	current alloy

## Data Availability

The data that support the findings of this study are available from the corresponding author, Yuyong Chen, upon reasonable request.

## References

[1] Dong S.L., Chen R.R., Guo J.J., Ding H.S., Su Y.Q., Fu H.Z. (2015). Microstructure and room temperature tensile property of as-cast Ti44Al6Nb1.0Cr2.0V alloy. Trans. Nonferrous Met. Soc. China.

[2] Shi C., Lu Z., Zhang K., Deng L., Wang C. (2018). Microstructure evolution and mechanical properties of γ-TiAl honeycomb structure fabricated by isothermal forging and pulse current assisted diffusion bonding. Intermetallics.

[3] Clemens H., Mayer S. (2013). Design, processing, microstructure, properties, and applications of advanced intermetallic TiAl alloys. Adv. Eng. Mater..

[4] Wang Q., Chen R., Yang Y., Guo J., Su Y., Ding H., Fu H. (2018). Effects of V and B, Y additions on the microstructure and creep behaviour of high-Nb TiAl alloys. J. Alloys Compd..

[5] Ping-Ping J. (2018). High-temperature Antioxidation of TiAl-Nb Based Alloys. Surf. Technol..

[6] Fan J., Liang L., Liu Z., Li Y., Li Y., Gao H., Wu S., Wang Y., Wang X. (2019). Recent research and development of mould materials for casting TiAl alloys. Mater. Sci. Technol..

[7] (2018). Microstructure stability and micro-mechanical behavior of as-cast gamma-TiAl alloy during high-temperature low cycle fatigue. Acta Mater..

[8] Liu C.T., Maziasz P.J., Clemens D.R., Kim Y.W., Wagner R., Yamaguchi M. (1995). Gamma Titanium Aluminides.

[9] Gerling R., Bartels A., Clemens H., Kestler H., Schimansky F.P. (2004). Structural characterization and tensile properties of a high niobium containing gamma TiAl sheet obtained by powder metallurgical processing. Intermetallics.

[10] Liu Y., Liang X., Liu B., He W., Li J., Gan Z., He Y. (2014). Investigations on processing powder metallurgical high-Nb TiAl alloy sheets. Intermetallics.

[11] Kawabata T., Tadano M., Izumi O. (1988). Effect of purity and second phase on ductility of TiAl. Scripta Metallurgica. Scr. Metall..

[12] Du Z., Zhang K., Lu Z., Jiang S. (2018). Microstructure and mechanical properties of vacuum diffusion bonding joints for γ-TiAl based alloy. Vacuum.

[13] Mathabathe M.N., Bolokang A.S., Govender G., Mostert R.J., Siyasiya C.W. (2018). The vacuum melted γ-TiAl (Nb, Cr, Si)-doped alloys and their cyclic oxidation properties. Vacuum.

[14] Hwang S.K. (2002). Microstructural refinement and improvement of mechanical properties and oxidation resistance in EPM TiAl-based intermetallics with yttrium addition. Acta Mater..

[15] Chen Y.Y., Li B.H., Kong F.T. (2008). Microstructural refinement and mechanical properties of Y-bearing TiAl alloys. J. Alloys Compd..

[16] Gourdet S., Montheillet F. (2000). An experimental study of the recrystallization mechanism during hot deformation of aluminium. Mater. Sci. Eng. A.

[17] Trivedi P.B., Patankar S.N., Froes F.S., Baburaj E.G., Genç A., Ovecoglu L. (2002). Grainsize control in Ti-48Al-2Cr-2Nb with yttrium additions. Metall. Mater. Trans. A.

[18] Criss J.W., Birks L.S., McKinley T.D., Heinrich K.F.J., Wittry D.B. (1966). Calibration of chemical element EDS. The Electron Microprobe.

[19] Appel F., Oehring M., Paul J.D.H. (2006). Nano-scale design of TiAl alloys based on β phase decomposition. Adv. Eng. Mater..

[20] Takeyama M., Kobayashi S. (2005). Physical metallurgy for wrought gamma titanium aluminides: Microstructure control through phase transformations. Intermetallics.

[21] Su Y., Kong F., Chen Y., Gao N., Zhang D. (2013). Microstructure and mechanical properties of large size Ti-43Al-9V-0.2Y alloy pancake produced by pack-forging. Intermetallics.

[22] Ke Y., Duan H., Sun Y. (2010). Effect of yttrium and erbium on the microstructure and mechanical properties of Ti–Al–Nb alloys. Mater. Sci. Eng. A.

[23] Kong F.T., Chen Y.Y., Tian J. (2004). Effect of Yttrium on Microstructure and Mechanical Properties of Ti-43Al-9V Alloy. Chin. J. Rare Met..

[24] Lu W.J., Xiao L., Xu D., Qin J.N., Zhang D. (2007). Microstructural characterization of Y2O3 in in situ synthesized titanium matrix composites. J. Alloys Compd..

[25] Zhang Y., Wang X., Kong F., Chen Y. (2018). A high-performance β-stabilized Ti-43Al-9V-0.2Y alloy sheet with a nano-scaled, antiphase domain. Mater. Lett..

[26] Li B., Kong F., Chen Y. (2006). Effect of Yttrium Addition on Microstructures and Room Temperature Tensile Properties of Ti-47 Al Alloy. J. Rare Earths.

[27] Chen Y.Y., Li B.H., Kong F.T. (2007). Effects of minor yttrium addition on hot deformability of lamellar Ti-45Al-5Nb alloy. Trans. Nonferrous Met. Soc. China.

[28] Chen Y., Niu H., Kong F., Xiao S. (2011). Microstructure and fracture toughness of a β phase containing TiAl alloy. Intermetallics.

[29] Trivedi P.B., Patankar S.N., Froes F.S., Baburaj E.G. (2002). Formation of Al–Y oxide during processing of γ-TiAl. J. Alloys Compd..

[30] Cui N., Kong F., Wang X., Chen Y., Zhou H. (2016). Microstructural evolution, hot workability, and mechanical properties of Ti–43Al–2Cr–2Mn–0.2Y alloy. Mater. Des..

[31] Zhou H., Kong F., Wu K., Wang X., Chen Y. (2017). Hot pack rolling nearly lamellar Ti-44Al-8Nb-(W, B, Y) alloy with different rolling reductions: Lamellar colonies evolution and tensile properties. Mater. Des..

[32] Zong Y., Wen D., Liu Z., Shan D. (2016). γ-Phase transformation, dynamic recrystallization and texture of a forged TiAl-based alloy based on plane strain compression at elevated temperature. Mater. Des..

[33] Kenel C., Dasargyri G., Bauer T., Colella A., Spierings A.B., Leinenbach C., Wegener K. (2017). Selective laser melting of an oxide dispersion strengthened (ODS) γ-TiAl alloy towards production of complex structures. Mater. Des..

[34] Li H., Long Y., Liang X., Che Y., Liu Z., Liu Y., Xu H., Wang L. (2020). Effects of multiaxial forging on microstructure and high temperature mechanical properties of powder metallurgy Ti-45Al-7Nb-0.3W alloy. Intermetallics.

[35] Zong Y., Wen D., Liu Z., Shan D. (2018). Molina-Aldareguia. Slip transfer across γ-TiAl lamellae in tension. Mater. Des..

[36] Erdely P., Staron P., Maawad E., Schell N., Klose J., Clemens H., Mayer S. (2017). Design and control of microstructure and texture by thermomechanical processing of a multi-phase TiAl alloy. Mater. Des..

[37] Bartels A., Hartig C., Uhlenhut H. (1997). Influence of the deformation conditions on the texture evolution in γ –TiAl. Mater. Sci. Eng. A.

[38] Kanani M., Hartmaier A., Janisch R. (2016). Stacking fault based analysis of shear mechanisms at interfaces in lamellar TiAl alloys. Acta Mater..

[39] Hang D.Y., Li H.Z., Liang X.P., Wei Z.W., Liu Y. (2014). Microstructure characteristic for high temperature deformation of powder metallurgy Ti–47Al–2Cr–0.2Mo alloy. Mater. Des..

[40] Zhang D.Y., Li H.Z., Liang X.P., Wei Z.W., Liu Y. (1999). Brittle-to-ductile transition temperature and its strain rate sensitivity in a two-phase titanium aluminide with near lamellar microstructure. J. Mater. Sci..

[41] Al-Samman T., Molodov K.D., Molodov D.A., Gottstein G., Suwas S. (2012). Softening and dynamic recrystallization in magnesium single crystals during c-axis compression. Acta Mater..

[42] Kong F., Xu X., Chen Y., Zhang D. (2012). Microstructure and mechanical properties of large size as-cast Ti–43Al–9V–0.2Y (at.%) alloy ingot from brim to centre. Mater. Des..

